# Meniscoplasty for stable osteochondritis dissecans of the lateral femoral condyle combined with a discoid lateral meniscus: a case report

**DOI:** 10.1186/1752-1947-5-434

**Published:** 2011-09-06

**Authors:** Hong-Chul Lim, Ji-Hoon Bae

**Affiliations:** 1 Department of Orthopaedic Surgery, Korea University College of Medicine, Guro Hospital, 97 Gurodonggil, Gurogu, Seoul, 152-703, Republic of Korea; 2 Department of Orthopaedic Surgery, Korea University College of Medicine, Ansan Hospital, Gojan 1 Dong, Danwon Gu, Ansan Si, Gyeonggi Do, 425-707, Republic of Korea

## Abstract

**Introduction:**

Osteochondritis dissecans of the lateral femoral condyle is relatively rare, and it is reported to often be combined with a discoid lateral meniscus. Given the potential for healing, conservative management is indicated for stable osteochondritis dissecans in patients who are skeletally immature. However, patients with osteochondritis dissecans of the lateral femoral condyle combined with a discoid lateral meniscus often have persistent symptoms despite conservative management.

**Case presentation:**

We present the case of a seven-year-old Korean girl who had osteochondritis dissecans of the lateral femoral condyle combined with a discoid lateral meniscus, which healed after meniscoplasty for the symptomatic lateral discoid meniscus without surgical intervention for the osteochondritis dissecans. In addition, healing of the osteochondritis dissecans lesion was confirmed by an MRI scan five months after the operation.

**Conclusions:**

Meniscoplasty can be recommended for symptomatic stable juvenile osteochondritis dissecans of the lateral femoral condyle combined with a discoid lateral meniscus when conservative treatment fails.

## Introduction

Osteochondritis dissecans (OCD) is a condition of the joints that appears to primarily affect subchondral bone, with secondary effects on articular cartilage. Initially, softening of the overlying articular cartilage is noted with an intact articular surface; this can progress to early articular cartilage separation, partial detachment of an articular lesion, and eventually osteochondral separation with a loose body. Etiologic theories of traumatic, ischemic, accessory ossification center persistence and various genetic factors have been proposed [1-5].

Several investigators have shown subsequently that there is an increased occurrence of OCD lesions of the lateral femoral condyle associated with a discoid lateral meniscus [6-9]. A discoid lateral meniscus might play an important role in causing OCD of the lateral femoral condyle among patients who are still growing. Repetitive abnormal stress on weaker osteochondral structures produced by a discoid meniscus during growth may cause OCD of the lateral femoral condyle. Given the potential for healing, conservative management is indicated for stable OCD in patients who are skeletally immature. However, patients with OCD of the lateral femoral condyle combined with a discoid lateral meniscus often have persistent symptoms despite conservative management [8,10].

We present a case of OCD of lateral femoral condyle combined with a discoid lateral meniscus, which healed after meniscoplasty for the symptomatic lateral discoid meniscus without surgical intervention for the OCD.

### Case presentation

A seven-year-old Korean girl presented with left knee pain of three months' duration. A physical examination demonstrated a five-degree extension block and tenderness on the lateral joint line. The result of a McMurray test was positive. An MRI scan revealed a complete discoid lateral meniscus with a bucket handle tear. On arthroscopy, a complete discoid lateral meniscus with longitudinal tear was found that extended throughout the entire meniscus. Subtotal meniscectomy with reshaping of remnant meniscus tissue was performed. Our patient had no further symptoms stemming from the torn meniscus and recovered a full range of motion. Activity was not restricted following recovery from the surgical intervention.

**Figure 1:**
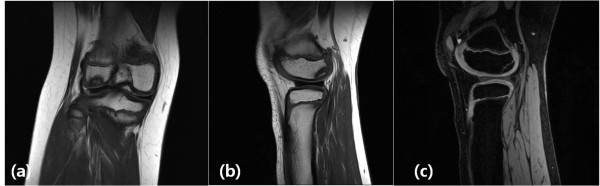
**(A, B) MRI study showing a discoid lateral meniscus with a 1.5 by 1.5 cm osteochondral lesion involving the posterior articular surface of the lateral femoral condyle**. (C) There was no evidence of fluid signal intensity between host and fragment on a T2-weighted MRI scan.

**Figure 2:**
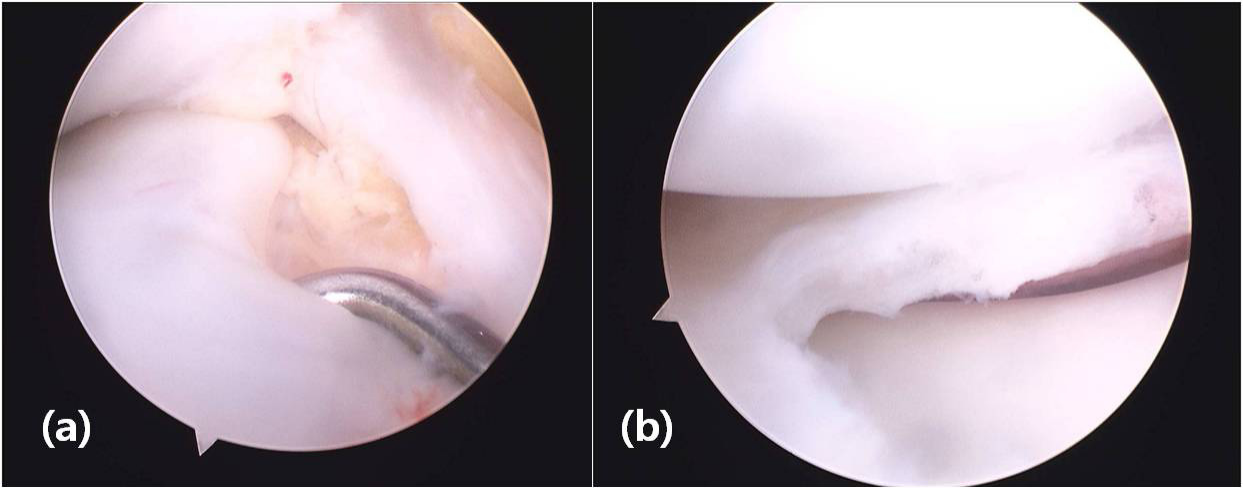
**(A) Arthroscopic picture showing a complete type of discoid lateral meniscus of right knee joint and (B) meniscoplasty with a stable 6 mm peripheral remaining meniscus**.

**Figure 3:**
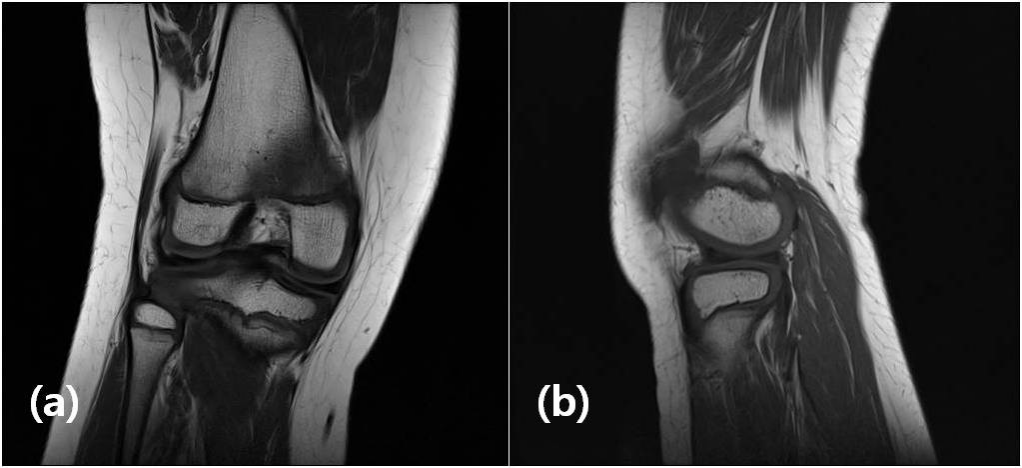
**Our patient five months after operation**. MRI study showing complete healing of the osteochondritis dissecans lesion of the lateral femoral condyle.

Two years after her first operation, our patient presented with a snapping sound and intermittent pain involving her right knee. A physical examination at this time revealed mild tenderness to the lateral joint line, but all other test results and findings from plain radiographs were normal. An MRI scan showed a complete discoid lateral meniscus with a 1.5 by 1.5 cm osteochondral lesion involving the posterior articular surface of the lateral femoral condyle (Figure 1A). There was no evidence of fluid signal intensity between the host and fragment on a T2-weighted MRI scan (Figure 1B). Initially, our patient was treated with conservative management consisting of activity modification. However, our patient had persistent symptoms despite six months of conservative management and she therefore underwent operation. On arthroscopy, a complete discoid lateral meniscus was identified (Figure 2A). The articular surface of the lateral femoral condyle had normal articular continuity and contour, but softening of cartilage at the margins of the OCD within the lateral femoral condyle without breach or fibrillation was found. We performed meniscoplasty that provided a stable 6 mm peripheral of the remaining meniscus and no treatment was performed for the OCD lesion (Figure 2B). Post-operatively, our patient was allowed to begin full weight bearing without immobilization and started a physical therapy protocol to improve the range of motion in her knee. Five months after the operation, an MRI scan demonstrated complete resolution of the previous OCD lesion of the lateral femoral condyle (Figure 3). There was no restriction of early activity following the surgical intervention. Our patient had no symptoms on either knee and had returned to full daily activity.

## Discussion

The findings in this case report may support the proposed etiology that a discoid lateral meniscus can produce repetitive abnormal stress on weaker osteochondral structures in the growing period, and may cause OCD of the lateral femoral condyle. Mitsuoka *et al. *[8] reported the case of a 10-year-old boy who was treated with partial meniscectomy for a discoid lateral meniscus without any treatment for OCD of the lateral femoral condyle. They suggested that an abnormal repetitive loading on weaker osteochondral structures by the damaged discoid lateral meniscus is considered to be one of the main causes of OCD of the lateral femoral condyle. Matsumoto *et al. *[10] reported a case with bilateral OCD lesions of the lateral femoral condyle in which the lesions were successfully healed by meniscoplasty. They proposed an abnormal contact force may lead to OCD lesion in the lateral femoral condyle. From these observations, our hypothesis is that correction of abnormal loading to the lateral femoral condyle by meniscoplasty can result in complete healing of an osteochondral lesion.

Non-surgical treatment including activity modification is primarily indicated for stable juvenile OCD. It may include crutches for limited weight bearing as well as braces or even casts for patients who are non-compliant. Gauzy *et al. *[11] followed a group of 30 children to complete resolution of symptoms by discontinuing sports activities. The authors recommended no surgical intervention because symptoms resolved with discontinuation of sports activities. However, there are concerns about the conservative treatment such as longer time to heal and the possibility of recurrence in cases of OCD of lateral femoral condyle combined with a discoid lateral meniscus. In addition, it is difficult to differentiate whether OCD or the discoid lateral meniscus is the cause of symptoms. In our patient's case, the OCD lesion healed and the symptoms improved immediately after meniscoplasty, while conservative treatment failed. It is difficult to conclude that healing of the OCD lesion was a result of meniscoplasty alone, and we cannot exclude the effect of activity modification or the natural healing process of stable OCD in a growing child. However, it is our belief that if the discoid lateral meniscus is combined with OCD in the lateral femoral condyle, there is a high possibility that conservative treatment will fail.

Arthroscopic drilling has been suggested for stable lesions with an intact articular surface [12-14]. Subchondral drilling creates channels to promote revascularization and healing. Several published papers have described cases of concomitant juvenile OCD of the lateral femoral condyle with discoid lateral meniscus [6-9]. Of those, only one published paper described subchondral bone drilling for an OCD lesion and reported satisfactory results [8]. In contrast, our patient's case showed that meniscoplasty without surgical intervention for the OCD lesion can lead to complete healing of the OCD lesion five months after the operation.

## Conclusions

Meniscoplasty can be recommended for symptomatic stable juvenile OCD of the lateral femoral condyle combined with a discoid lateral meniscus when conservative treatment fails.

## Consent

Written informed consent was obtained from the patient's next-of-kin for publication of this case report and any accompanying images. A copy of the written consent is available for review by the Editor-in-Chief of this journal.

## Competing interests

The authors declare that they have no competing interests.

## Authors' contributions

HCL performed the operation and was a major contributor to writing the manuscript. JHB also helped write the manuscript and assisted with the figures.
